# EHMTI-0266. Validation of a sham manipulative procedure: a new protocol for conducting placebo-control clinical trials in manual therapy

**DOI:** 10.1186/1129-2377-15-S1-E7

**Published:** 2014-09-18

**Authors:** A Chaibi, J Saltyte Benth, P Tuchin, MB Russell

**Affiliations:** 1 Head and Neck Research Group, University of Oslo and Reserach Centre Akershus University Hospital, Lorenskog, Norway; 2 HØKH Research Centre, Institute of Clinical Medicine Akershus University Hospital, Lorenskog, Norway; 3 Department of Chiropractic, Macquarie University, Sydney, Australia; 4 Head and Neck Research Group, Research Centre Akershus University Hospital, Lorenskog, Norway

## Background

Few manual therapy studies have attempted to conduct placebo-control clinical trials. Thus, quantification of alleged placebo effects consequently becomes difficult.

## Aim

To investigate and validate a new placebo intervention for spinal manipulative therapy clinical trials, i.e. sham manipulation, and investigate the feasibility of a short de-blinding questionnaire.

## Method

A single blinded, prospective randomized, placebo-controlled trial with 1 month baseline and 3 months treatment with 12 treatments. 104 participants diagnosed with migraine were equally randomized into 1 of 3 groups: (i) chiropractic spinal manipulative therapy (CSMT), (ii) placebo (sham manipulation), (iii) control group (continued usual management). The participants filled in questionnaire on de-blinding after each treatment session. Primary end-point was the rate of successful blinding through de-blinding questionnaires given after each treatment session.

## Results

772 out of 840 individual cases were analyzed, only 8.1 % out of all cases missed their appointment. The unadjusted result shows that both the active and placebo group believed they received active treatment with odds ratio (OR) of ≥88.9 and ≥80.0 respectively. Due to strong cluster effect in our data, logistic regression model was used to adjust for repeated measures which showed a significant OR of >10 in both the active and the placebo group.

## Conclusion

This is the first study to successfully demonstrate a manipulative sham procedure over a full length treatment period, assessing the placebo group with de-blinding questionnaire after each session which thus, could be incorporated in future clinical trials.

No conflict of interest.

